# Factors affecting trough concentrations of voriconazole: a dual-center retrospective analysis focusing on loading dose and inflammatory state effects

**DOI:** 10.3389/fphar.2026.1745737

**Published:** 2026-04-23

**Authors:** Feng Chen, Qiong Liu, Jing Wen, Lin Hu, Qi Huang, Yueping Jiang

**Affiliations:** 1 Department of Clinical Pharmacy, Hunan University of Medicine General Hospital, Huaihua, China; 2 National Clinical Research Center for Geriatric Disorders, Xiangya Hospital, Central South University, Changsha, Hunan, China; 3 Department of Oncology, Xiangya Hospital of Central South University, Changsha, Hunan, China; 4 Department of Pharmacy, The Third People’s Hospital of Yunnan Province, Kunming, Yunnan, China; 5 Department of Pharmacy, The First Hospital of Changsha, Changsha, Hunan, China; 6 Department of Pharmacy, Xiangya Hospital, Central South University, Changsha, Hunan, China; 7 College of Pharmacy, Changsha Medical University, Changsha, Hunan, China

**Keywords:** C-reactive protein, inflammatory, loading dose, TDM, voriconazole

## Abstract

**Background:**

There are significant interindividual variations in voriconazole (VCZ) blood concentrations, which affect both treatment efficacy and safety.

**Objective:**

Our goal is to identify the factors influencing VCZ trough concentrations and provide new evidence for individualized dosing.

**Methods:**

A total of 281 hospitalized patients receiving VCZ were enrolled. Demographic, liver and kidney function, C-reactive protein (CRP) and other clinical data were collected. Trough concentrations were determined using HPLC. Multivariate linear regression and ordinal logistic regression were employed to identify influencing factors, with predictive performance assessed by ROC curves.

**Results:**

CRP is a significant positive predictor of VCZ trough concentration (B = 0.010, *p* = 0.006), demonstrating the strongest predictive capability for elevated trough levels (>5 μg/mL) (AUC = 0.8864, *p* < 0.0001), outperforming conventional liver function indicators such as total bile acids (AUC = 0.6326). Loading dose also showed a significant correlation with increased trough concentration (B = 0.973, *p* = 0.007) and elevated the risk of supratherapeutic levels (OR = 0.430, *p* = 0.006). Additionally, weight, albumin, platelet count, and concomitant administration of pantoprazole or dexamethasone were identified as independent influencing factors.

**Conclusion:**

The high variability in VCZ trough concentrations may be partially attributed to factors such as weight, loading dose, liver function, inflammation and concomitant medications. Although loading doses enable rapid efficacy, they increase the risk of supratherapeutic concentrations. Patients in the high-CRP group are more likely to exceed 5.0 μg/mL, demonstrating the best discriminative ability for predicting excessively high concentrations. These findings provide new evidence for VCZ dose optimization.

## 1 Introduction

Voriconazole (VCZ) is a broad-spectrum antifungal medication and is the first-line treatment for invasive aspergillosis. Multiple studies have shown that the trough concentration of VCZ is closely related to its efficacy and safety (Dolton and McLachlan, 2014). After absorption, there is significant interindividual variability in serum drug concentrations (Schulz et al., 2019). When the trough concentration of VCZ is low (below 1.0 mg/L), it may lead to treatment failure; conversely, when the trough concentration is too high (above 5.0 mg/L), it increases the risk of toxicity, including hepatotoxicity, neurotoxicity, visual disturbances, and rashes (Luong et al., 2016). Developing individualized treatment plans is an important research direction in the field of pharmacy, aiming to provide more personalized and precise dosing regimens based on individual patient characteristics and disease conditions.

VCZ exhibits nonlinear pharmacokinetic characteristics in adults, with its serum concentration rising significantly as the dosage increases. To achieve effective therapeutic concentrations quickly, current guidelines generally recommend the use of a loading dose (Takesue et al., 2022). However, there is still insufficient research evidence regarding whether a loading dose may lead to serum concentrations exceeding the safe range. VCZ is primarily metabolized by cytochromeP450 (CYP450) 2C19, with secondary pathways including CYP3A4 and CYP2C9. Additionally, it is a strong inhibitor of CYP3A4, meaning that any factors that may affect liver enzyme activity could be related to its serum concentration (Niece et al., 2015). Existing studies have indicated that factors such as age, CYP2C19 genetic polymorphisms, liver function indicators (such as transaminases, albumin, bilirubin, etc.), and the concomitant use of liver enzyme inhibitors or inducers may alter the absorption, distribution, metabolism and excretion processes of VCZ, thereby affecting its serum concentration (Hu et al., 2023; Zh et al., 2021). Although these influencing factors are widely recognized, inconsistencies often arise in the existing research results due to the complexity and dynamic nature of physiological and pathological processes. This suggests that there may still be unidentified factors influencing VCZ serum concentrations that warrant further investigation.

Recent studies have shown that infections or inflammatory responses can lead to the downregulation of various CYP450 enzymes, and the reduced activity of these enzymes may decrease the hepatic clearance of VCZ (Gautier-Veyret et al., 2021). C-reactive protein (CRP), a commonly used clinical inflammatory marker, can effectively reflect the severity and progression of the body’s inflammatory response (Encalada Ventura et al., 2015; Vreugdenhil et al., 2018). Multiple studies have reported a positive correlation between CRP levels and the trough concentrations of VCZ. However, significant heterogeneity in patient populations, underlying disease states, CYP2C19 genotype distributions and concomitant medications across these studies presents challenges for direct comparison and generalization of results (Encalada Ventura et al., 2015; Vreugdenhil et al., 2018; Ling et al., 2024). For example, some studies focused on patients with hematological disorders or pediatric populations, who often have more complex clinical features and comorbidities, further complicating the analysis (Zh et al., 2021; Hu et al., 2025). Additionally, many existing studies are single-center designs or have insufficient sample sizes, limiting their ability to adequately control for potential confounding factors and affecting the robustness of their conclusions. Therefore, more high-quality, large-sample studies are needed to clarify the actual value of CRP in the individualized use of VCZ.

Therefore, this study employs a dual-center, retrospective research design to comprehensively analyze the effects of factors such as age, sex, weight, dosing regimens, liver and kidney function, complete blood count, concomitant medications, and inflammation on the trough concentrations of VCZ, providing more valuable guidance for clinical practice.

## 2 Materials and methods

### 2.1 Patients and study design

We conducted a retrospective analysis of the medical records of patients hospitalized between 1 January 2023, and 31 December 2024, at Xiangya Hospital of Central South University and Hunan University of Medicine who received VCZ treatment. We measured their steady-state trough concentrations (C_min_). This study was conducted in strict accordance with the Declaration of Helsinki and received approval from the Ethics Committee of Xiangya Hospital of Central South University or Hunan University of Medicine.

Inclusion criteria: (1) Administration regimen involving oral voriconazole 0.2 g twice daily, with a loading dose of 0.4 g when applicable; (2) Patients treated with a loading dose regimen with maintenance therapy lasting more than 2 days; (3) Patients without a loading dose but with maintenance therapy lasting more than 3 days.

Exclusion criteria: (1) Pregnant women; (2) Individuals under 18 years of age; (3) Patients with incomplete information; (4) Patients who had not reached steady-state concentrations at the time of blood sample collection or were undergoing blood purification treatment. (5) Patients with known CYP2C19 loss-of-function homozygous genotypes or those carrying rare genotypes associated with dual inhibition of CYP2C19 and CYP3A4 (if explicitly documented in clinical records).

### 2.2 Data collection

Clinical data of the patients were collected through the electronic medical record system, including demographic information (such as age, sex, and weight), as well as liver function indicators (Alanine Aminotransferase (ALT), Aspartate Aminotransferase (AST), Albumin (ALB), Globulin (GLB), Direct Bilirubin (DBIL), Indirect Bilirubin (IBIL), Total Bile Acids (TBA)), kidney function indicators (Urea (UREA), Serum Creatinine (CREA-S), Uric Acid (UA)), complete blood count (White Blood Cell Count (WBC), Hemoglobin (HGB), Platelet Count (PLT), Red Blood Cell Count (RBC), Neutrophil Count (NEU)), CRP levels and data on concomitant medications (According to literature reports, these agents may affect CYP450 enzyme activity or the pharmacokinetics of VCZ, we ultimately included omeprazole, pantoprazole, budesonide, dexamethasone, methylprednisolone, isoniazid, and rifampicin in the analysis.). This study required that the CRP measurements used for analysis be collected on the same day as the steady-state trough concentration sampling for VCZ or within the immediately preceding day (with an interval of no more than 24 h). If a patient underwent multiple CRP tests on the same day, the measurement closest in time to the VCZ blood sampling was selected.

### 2.3 Total trough concentration of VCZ in serum of determination

#### 2.3.1 Administration regimen and blood collection

The standard loading dose regimen (400 mg every 12 h) is expected to achieve steady-state VCZ serum concentrations after at least 2 consecutive days of administration, whereas the maintenance dose regimen without a loading dose (200 mg every 12 h) requires at least 3 consecutive days to reach steady state. To ensure that the measured concentrations represent steady-state trough levels, all blood samples were collected at the end of the dosing interval (i.e., within 30 min before the next dose) after VCZ had reached steady state. Blood samples were collected using red serum separator tubes (containing inert separation gel and clot activator), with a volume of 2–3 mL per sample. The tubes were immediately inverted and mixed 5–8 times after collection.

#### 2.3.2 Chromatographic test

The method used in this study is high-performance liquid chromatography (HPLC) for detecting the trough concentration of VCZ in the blood. Reagents: VCZ (China National Food and Drug Administration, batch number: 100862–201903), Estazolam (China National Food and Drug Administration, batch number: 171219–201003), Methanol (chromatographic purity, Anhui Tiandi High-purity Solvent Co., Ltd.) and purified water (prepared by Xiangya Hospital of Central South University); Chromatographic conditions: The chromatographic system, from Shimadzu, consisted of a LC-20 A pump, equipped with a 100 μL injection loop, and a programmable SPD-10 Av UV-vis absorbance detector. Separation for VCZ was performed by Agilent Zorb Ax 3B-C18 (250 × 4.6 mm, 5 μm) analytical column with a mobile phase consisting of methanol: water (60: 40, v/v) set at a flow rate of 1.0 mL/min. The UV detection was set at 256 nm. The retention time of VCZ and internal standard (estazolam) was found to be 9.6 and 8.2 min respectively. Standard curve: 195 µL blank plasma was precision added with 5 µL of VCZ reference solution, and a series of plasma samples were prepared with different concentrations. According to the operation of blood sample pretreatment, the peak area ratio of estazolam was used to regress the concentration of VCZ in plasma samples, and a regression equation was established (Y = 1.2980 X + 0.0031, r = 0.9998). Then, 200 µL plasma was accurately sucked into a 2 mL EP tube, 10 μL of internal standard estazolam solution (50 mg/L) was added and vortex mixed for 30 s. Add 1,600 μL of mixed extract (N-hexane 1,200 μL and ethyl acetate 400 μL), vortex mixing for 1 min and centrifugation at 14500 *g* for 8 min. Then, 1,400 μL supernatant was sucked into another EP tube, and volatilized at N_2_ for 20 min. Then it was reconstituted with 50% methanol (150 μL), vortex-mixed for 30 s, and centrifuged at 14500 *g* for 3 min. Transfer 100 μL into the liner tube with a pipette, put the sample into the HPLC rack, and analyze it with the injection volume of 20 μL.

The linear range of the method was 0.313∼28.55 mg/L (Y = 1.2980 X + 0.0031, r = 0.9998). LLOQ was 0.313 mg/L, and S/N > 10. The precision, accuracy, matrix effect, recovery rate and stability meet the requirements of the bioanalysis method validation guidelines (summarized in Supplementary Tables S1-S3; Supplementary Figures S1-S3). The measured concentrations of all clinical samples fell within the linear range of the standard curve (0.313–28.55 mg/L).

### 2.4 Statistical analysis

Data analysis was conducted using SPSS 22.0 statistical software (SPSS Inc., Chicago, Illinois, USA) and GraphPad software (GraphPad Prism 7.0) was used for plotting. The Kolmogorov-Smirnov test was employed to assess normality. Continuous variables that followed a normal distribution were expressed as means and standard deviations (SD), and comparisons between groups were performed using independent samples t-tests and one-way ANOVA. Non-normally distributed continuous variable data were expressed as medians and interquartile ranges (IQR), and non-parametric tests were used (Mann-Whitney U test for two groups or Kruskal-Wallis test for at least three groups). Categorical variables were presented as frequencies and percentages, and comparisons were made using the χ^2^ test or Fisher’s exact test. Spearman correlation was used to assess the relationship between VCZ concentrations and other continuous variables. Variables with p ≤ 0.10 were included in multiple linear regression analysis to identify independent factors affecting VCZ trough concentrations. The relationship between independent influencing factors and VCZ serum concentrations was evaluated using receiver operating characteristic (ROC) curves. Variables with p ≤ 0.10 from univariate logistic regression analysis were included in generalized ordered multiple logistic regression analysis to explore independent factors affecting the achievement of target VCZ trough concentrations. A p-value of <0.05 was considered statistically significant.

## 3 Result

### 3.1 Patient characteristics

A total of 281 patients were included in this study after screening. Among them, 191 were male (68.0%) and 90 were female (32.0%), with a median age of 64 years (interquartile range, IQR: 55–73.5). Most patients were over 65 years old (48.4%). The median weight was 56 kg (IQR: 49–64), and the median daily dose of VCZ was 0.4 g. A total of 175 patients (65.1%) received loading dose therapy. The median trough concentration (Cmin) of VCZ was 3.44 μg/mL (IQR: 2.04–5.26). Among these, 23 patients (8.2%) had concentrations below the therapeutic window (<1 μg/mL), 180 patients (64.0%) were within the therapeutic window (1–5 μg/mL), and 78 patients (27.8%) had concentrations above the therapeutic window (>5 μg/mL). Concomitant medications mainly included omeprazole, pantoprazole, budesonide, dexamethasone, methylprednisolone, and rifampicin, among others. The most common underlying conditions were hypertension, anemia, diabetes, and leukemia (Table 1).

**TABLE 1:** Basic information of patients.

Characteristics	Value	Characteristics	Value
Total number of patients (n)	281	CRP (40–200 mg‧L^–1^)	40.5 (12.0, 97.3)
Sex [n (%)]		Combination drugs, n (%)	
Male	191 (68.0)	Pantoprazole	82 (29.2)
Female	90 (32.0)	Omeprazole	51 (18.1)
Age (years)	64 (55, 73.5)	Dexamethasone	21 (7.5)
18–45 years n (%)	19 (6.8)	Budesonide	77 (27.4)
46–65 years n (%)	126 (44.8)	Atorvastatin	34 (12.1)
>65 years n (%)	136 (48.4)	Methylprednisolone	35 (12.5)
Weight (Kg)	56 (49 64)	Isoniazid	14 (5.0)
Daily dose (g)	0.4 (0.4, 0.4)	Rifampicin	7 (2.5)
Loading dose strategy, n (%)		Underlying disease, n (%)	
None loading dose	94 (34.9)	Hypoalbuminemia	31 (11.0)
Loading dose	175 (65.1)	Hypertension	63 (22.4)
ALT (7–40 U‧L^-1^)	22 (13, 38)	Diabetes	11 (3.9)
AST (5–40 U‧L^-1^)	30 (21, 47)	Renal insufficiency	25 (8.9)
ALB (40–55 g‧L^-1^)	31.25 (27.5, 35.9)	Novel coronavirus infection	19 (6.8)
GLB (20–40 g‧L^-1^)	27.95 (24.1, 33.1)	Anemia	29 (10.3)
DBIL (0–8.6 μmoL‧L^-1^)	3.2 (2.05, 5.2)	Granulocytopenia	7 (2.5)
IBIL (3–15 μmoL‧L^-1^)	4.1 (3, 5.8)	Thrombocytopenia	5 (1.8)
TBA (0–12 μmoL‧L^-1^)	4.1 (2.5, 7.7)	Leukopenia	7 (2.5)
UREA (2.6–7.5 mmoL‧L^-1^)	6.175 (4.3, 10.3)	Severe pneumonia	23 (8.2)
CREA-S (57–97 μmoL‧L^-1^)	72.5 (58, 104)	Sepsis	8 (2.8)
UA (150–350 μmoL‧L^-1^)	219 (163, 322)	HIV	5 (1.8)
WBC (3.5–9.5 10^9^‧L^-1^)	6.8 (4.6, 9.2)	Tuberculosis	15 (5.3)
HGB (110–150 g‧L^-1^)	93 (72.5, 110)	VCZ C_SS min_ (μg‧mL^–1^)	3.44 (2.04, 5.26)
PLT (125–350 10^9^‧L^-1^)	202 (114, 279)	<1 μg‧mL^–1^, n (%)	23 (8.2)
RBC (3.5–5 10^12^‧L^-1^)	3.13 (2.4, 3.8)	1–5 μg‧mL^–1^, n (%)	180 (64.0)
NEU (1.80–6.3 10^9^‧L^-1^)	4.83 (2.8, 7.3)	>5 μg‧mL^–1^, n (%)	78 (27.8)

Continuous variables are presented as median (interquartile range), and categorical data are presented as percentages; ALT, Alanine Aminotransferase; AST, Aspartate Aminotransferase; ALB, Albumin; GLB, Globulin; DBIL, Direct Bilirubin; IBIL, indirect bilirubin; TBA, total bile acids; UREA, urea; CREA-S, serum creatinine; UA, uric acid; WBC, white blood cell count; HGB, hemoglobin; PLT, platelet count; RBC, Red Blood Cell Count; NEU, neutrophil count; HIV, human immunodeficiency virus; VCZ, voriconazole; CSS, min:Steady-state trough concentration.

### 3.2 Analysis of factors affecting voriconazole trough concentrations

**FIGURE 1:**
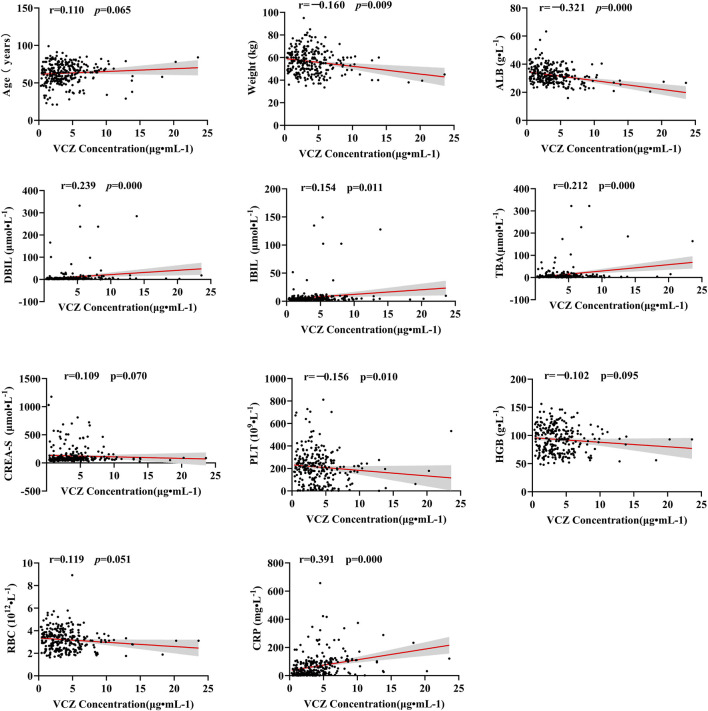
Correlation analysis of voriconazole trough concentration.

Spearman correlation analysis was conducted to assess the relationship between continuous variables such as daily dose, age, weight, liver and kidney function, complete blood count, and CRP with VCZ trough concentrations. The results indicated significant correlations between VCZ trough concentrations and several continuous variables. Specifically, direct bilirubin, indirect bilirubin, total bile acids, and CRP showed significant positive correlations with VCZ (DBIL: r = 0.239, *p* < 0.001; IBIL: r = 0.154, *p* < 0.05; TBA: r = 0.212, *p* < 0.001; CRP: r = 0.391, *p* < 0.001). Age and serum CREA-S exhibited a weak positive correlation (Age: r = 0.110, *p* = 0.065; CREA-S: r = 0.109, *p* = 0.070). Weight, ALB and PLT were negatively correlated with VCZ (Weight: r = −0.160, *p* = 0.009; ALB: r = −0.321, *p* < 0.001; PLT: r = −0.156, *p* = 0.01), while HGB and RBC also showed weak negative correlations (HGB: r = −0.102, *p* = 0.095; RBC: r = −0.119, *p* = 0.051) (Figure 1).

**FIGURE 2:**
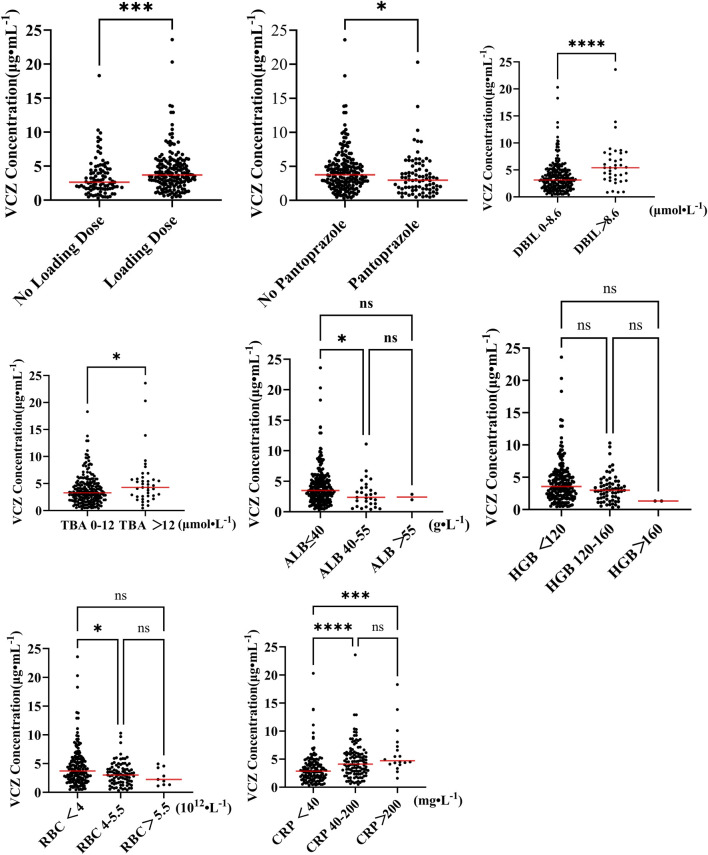
Comparison of VCZ trough concentrations between groups. (*p < 0.05, **p < 0.01, ***p < 0.001, ****p < 0.00001, ns *p* > 0.05).

Mann-Whitney U test showed that the use of loading doses (Z = −3.304, *p* = 0.001) and the concomitant use of pantoprazole (Z = −2.427, *p* = 0.015) were significantly associated with VCZ trough concentrations. Other concomitant medications, such as omeprazole and dexamethasone (Z = −1.886, *p* = 0.059), did not show a significant effect. Additionally, VCZ trough concentrations were analyzed by grouping based on age, CRP levels, and normal ranges of related biochemical indicators to assess differences among the groups. The Mann-Whitney U test for DBIL and TBA, divided into two groups, also indicated significant differences (DBIL: Z = −4.013, *p* < 0.001; TBA: Z = −2.231, *p* = 0.026). The Kruskal-Wallis test, applied to three groups, showed significant differences in ALB (H = 7.247, *p* = 0.027), HGB (H = 8.223, *p* = 0.016), RBC (H = 9.265, *p* = 0.01) and CRP (H = 30.034, *p* < 0.001) in relation to VCZ trough concentrations, but significant differences were found only among the ALB, RBC and CRP groups (Figure 2).

Using VCZ trough concentrations as the dependent variable, factors selected through univariate analysis with *p* < 0.1 included weight, use of a loading dose, concomitant use of pantoprazole, concomitant use of dexamethasone, ALB, DBIL (noting that DBIL and indirect bilirubin (IBIL) exhibited multicollinearity with a variance inflation factor (VIF) >5, so DBIL was chosen as the independent variable for regression analysis), TBA, HGB, PLT, RBC, CRP and serum CREA-S. Multiple linear regression analysis (*R* ^*2*^ = 0.270) identified several independent predictors of VCZ trough concentrations. Significant negative predictive factors included weight (B = −0.065, *p* < 0.001), ALB (B = −0.115, *p* = 0.001), the use of pantoprazole (B = −0.813, *p* = 0.035), and the use of dexamethasone (B = −1.400, *p* = 0.031). Positive predictive factors included the use of a loading dose (B = 0.973, *p* = 0.007), TBA (B = 0.023, *p* = 0.002) and CRP (B = 0.01, *p* = 0.006) (Table 2).

**TABLE 2:** Multiple linear regression analysis of factors influencing VCZ trough concentration.

Variable	Β	SE	β	95% Cl for Β	*p*-value	VIF
Age	0.010	0.013	0.041	[–0.017, 0.036]	0.475	1.142
Weight	−0.065	0.017	−0.220	[–0.099, −0.032]	0.000	1.128
Loading dose	0.973	0.359	0.150	[0.265, 1.68]	0.007	1.047
ALB	−0.115	0.035	−0.229	[–0.183, −0.047]	0.001	1.634
DBIL	−0.008	0.007	−0.097	[–0.023, 0.006]	0.255	2.493
TBA	0.023	0.007	0.270	[0.009, 0.037]	0.002	2.472
PLT	−0.002	0.001	−0.086	[–0.004, 0.001]	0.146	1.176
CRP	0.010	0.004	0.177	[0.003, 0.017]	0.006	1.379
Pantoprazole	−0.813	0.383	−0.121	[–1.568, −0.058]	0.035	1.104
Dexamethasone	−1.400	0.645	−0.123	[–2.67, −0.129]	0.031	1.088
HGB	0.008	0.008	0.064	[–0.008, 0.025]	0.317	1.384
RBC	−0.002	0.032	−0.004	[–0.065, 0.060]	0.943	1.026

Β unstandardized regression coefficient; SE, standard error; β standard regression coefficient; CI, confidence interval; VIF, variance inflation factor.

### 3.3 Factors influencing the achievement of target voriconazole trough concentrations

**FIGURE 3:**
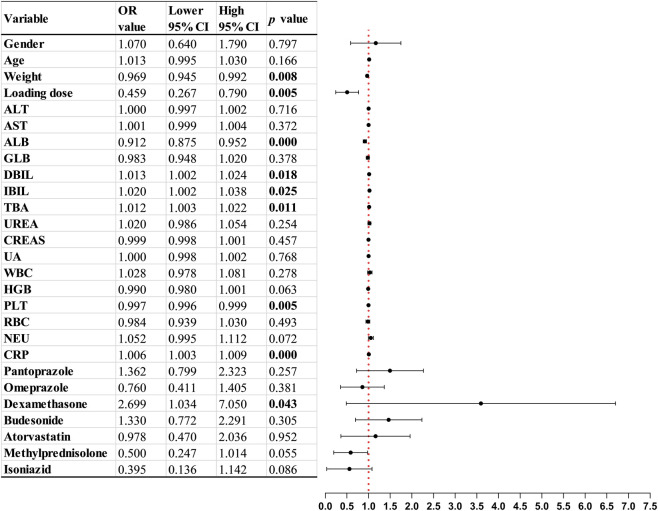
Univariate analysis results of VCZ trough concentration target achievement (*p < 0.05).

VCZ trough concentrations were categorized into three ordered levels: below the therapeutic window (<1 μg/mL), within the therapeutic window (1–5 μg/mL), and above the therapeutic window (>5 μg/mL). Logistic regression analysis was conducted to examine the influence of various factors on whether VCZ trough concentrations met the target levels. For variables that passed the parallelism test, univariate ordered logistic regression analysis was used. For variables that did not meet the parallelism assumption, generalized univariate ordered logistic regression analysis was applied. The results indicated that multiple factors were significantly associated with VCZ trough concentrations being below or above the therapeutic window, including weight (OR = 0.969, *p* = 0.008), use of a loading dose (OR = 0.459, *p* = 0.005), ALB (OR = 0.912, *p* < 0.001), DBIL (OR = 1.013, *p* = 0.018), TBA (OR = 1.012, *p* = 0.011), PLT (OR = 0.997, *p* = 0.005) and CRP (OR = 1.006, *p* < 0.001) (Figure 3).

Ordered multivariate logistic regression analysis further validated the impact of the aforementioned factors (Table 3). The model met the proportional odds assumption (*p* > 0.05), indicating that the effects of the independent variables were consistent across the different levels. The analysis results showed that multiple factors were independent predictors of VCZ trough concentration levels. Weight and PLT were significant negative predictors of VCZ trough concentrations (Weight: Β = −0.045, *p* = 0.002; PLT: Β = −0.003, *p* = 0.002). CRP was identified as a significant positive predictor (Β = 0.006, *p* = 0.028). Compared to patients who received a loading dose, those who did not have a significantly lower likelihood of achieving higher VCZ trough concentrations (Β = −0.844, *p* = 0.006). In contrast, patients who did not use dexamethasone had a significantly increased likelihood of achieving higher VCZ trough concentrations compared to those who did use it (Β = 1.307, *p* = 0.014). Although the effect of isoniazid (*p* = 0.065) did not reach statistical significance, its coefficient was negative, suggesting a potential trend toward reduced concentrations in patients not using isoniazid.

**TABLE 3:** Factors influencing the achievement of target VCZ trough concentration.

Variable	Β	SE	OR	95% Cl for OR	*p*-value
Weight	−0.045	0.014	0.956	[0.931, 0.983]	0.002
ALB	−0.048	0.028	0.953	[0.902, 1.006]	0.081
DBIL	0.009	0.009	1.009	[0.991, 1.027]	0.330
IBIL	−0.018	0.020	0.982	[0.946, 1.02]	0.353
TBA	0.010	0.007	1.010	[0.995, 1.024]	0.183
PLT	−0.003	0.001	0.997	[0.994, 0.999]	0.002
CRP	0.006	0.003	1.006	[1.001, 1.012]	0.028
HGB	0.001	0.007	1.001	[0.987, 1.014]	0.933
NEU	0.044	0.039	1.045	[0.968, 1.129]	0.266
No loading dose	−0.844	0.305	0.430	[0.236, 0.783]	0.006
Not combined with methylprednisolone	−0.617	0.434	0.540	[0.231, 1.262]	0.155
Not combined with isoniazid	−1.175	0.636	0.309	[0.089, 1.074]	0.065
Not combined with dexamethasone	1.307	0.532	3.695	[1.302, 10.486]	0.014

Β logistic regression coefficient; SE, standard error; OR, odds ratio; CI, confidence interval.

### 3.4 ROC curve analysis of predictive factors

**FIGURE 4:**
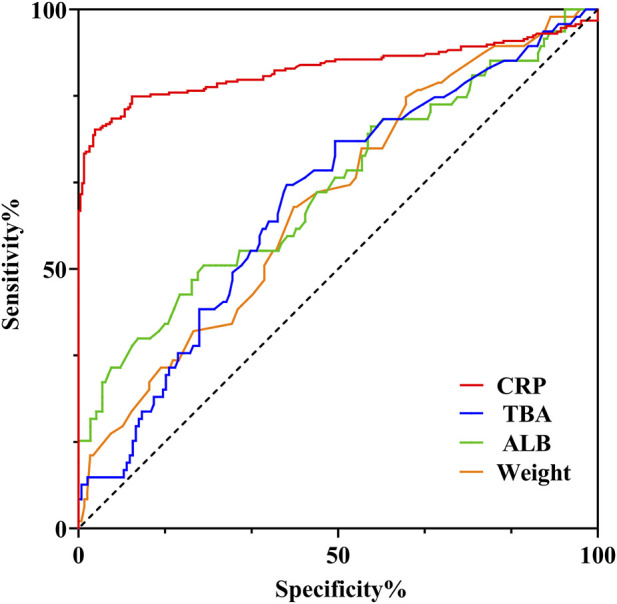
Prediction of VCZ concentration >5.0 μg/mL receiver operating characteristic curve based on CRP, ALB, TAB and Weight.

ROC curve analysis was used to evaluate the performance of various continuous variables in predicting whether the VCZ plasma concentration reached the target level (Figure 4). CRP demonstrated excellent predictive ability (AUC = 0.886, 95% CI: 0.855–0.918, p < 0.0001). The optimal cut-off value for predicting voriconazole trough concentrations >5 μg/mL was 89.1 mg/L, corresponding to a sensitivity of 55.3% and a specificity of 78.7%. In contrast, ALB (AUC = 0.6556, 95% CI = 0.5759∼0.7353, p = 0.0001), TBA (AUC = 0.6326, 95% CI = 0.5563∼0.7089, p = 0.0012) and weight (AUC = 0.6297, 95% CI = 0.5541∼0.7054, p = 0.0014), while statistically significant (all p-values <0.005), exhibited low AUC values, indicating limited practical discriminatory power. In summary, CRP is a strong predictor of VCZ concentration, whereas the predictive value of the other indicators may be limited.

## 4 Discussion

This dual-center, retrospective study analyzed clinical data from 281 patients treated with VCZ to systematically investigate the influence of various factors on the steady-state trough concentration of VCZ and identified independent predictors such as CRP, weight, loading dose and concomitant use of dexamethasone. The findings provide more detailed references for personalized dosing of VCZ.

Both multiple linear regression and ordinal logistic regression analyses in this study consistently identified CRP as a significant positive predictor of VCZ trough concentration (B = 0.010, p = 0.006; OR = 1.006, p = 0.028). This validates the classical theory that an inflammatory state inhibits metabolism, leading to increased drug concentrations (Hao et al., 2024). Although recent literature has addressed the relationship between inflammation and VCZ concentration—for instance, Klomp et al. suggested that inflammation alters the association between CYP2C19 genotype and VCZ activity, leading to phenotypic conversion (Klomp et al., 2024), and the study by Maeda Y et al. further discussed the relationship between inflammation and VCZ metabolism (Maeda et al., 2022). However, this study demonstrated through ROC curve analysis that CRP predicted VCZ trough concentrations >5 μg/mL with an AUC of 0.886. The optimal cut-off value was 89.1 mg/L, corresponding to a sensitivity of 55.3% and a specificity of 78.7%. This threshold indicates that patients with elevated CRP have an increased risk of supratherapeutic concentrations (specificity 78.7%), warranting enhanced monitoring. However, the relatively low sensitivity suggests that nearly half of the patients with concentrations exceeding the therapeutic range may be missed, which could be attributable to heterogeneity in inflammatory etiology, concomitant medication use, and lack of genotype data. Therefore, CRP should serve as one component of risk stratification rather than the sole decision-making criterion. For patients with CRP below the threshold but presenting other high-risk factors such as low body weight or hepatic insufficiency, vigilance remains warranted with timely implementation of TDM.

This study, through multiple linear regression analysis, revealed that loading dose administration was significantly associated with elevated VCZ trough concentrations (B = 0.973, p = 0.007). This strongly validates the consensus in both domestic and international guidelines and literature that loading doses are crucial for rapidly achieving therapeutic concentrations (Takesue et al., 2022). Concurrently, ordinal logistic regression analysis demonstrated that patients who did not receive a loading dose had a significantly reduced risk (57.0%) of trough concentrations >5 μg/mL (OR = 0.430, p = 0.006). This represents an exceptionally important and insightful finding. While previous studies have predominantly emphasized the positive role of loading doses in rapid target attainment, they have insufficiently addressed the “protective” risk mitigation against supratherapeutic levels (Zeng et al., 2020). Our findings clearly indicate that omitting the loading dose constitutes a strong risk factor for subtherapeutic concentrations, whereas conversely, its administration may also serve as a risk factor for elevated levels. As VCZ itself is an inhibitor of the CYP2C19 enzyme, the administration of a loading dose may exert an additional inhibitory effect on the enzyme, potentially leading to delayed metabolism (Lee et al., 2021). This suggests that in clinical practice, for patients with high metabolic risks (such as CYP2C19 poor metabolizers, or those with comorbid liver disease or significant inflammation), greater caution should be exercised when deciding to administer loading doses. Consideration should be given to implementing earlier TDM to avoid potential toxicity from excessive dosing. This perspective aligns with the findings of Guangting Zeng et al., who emphasized the high pharmacokinetic variability of VCZ and the necessity for optimized treatment regimens (Zeng et al., 2020).

Furthermore, factors such as weight, ALB, PLT and concomitant use of pantoprazole or dexamethasone were identified as negative predictors of VCZ trough concentrations. This reflects the complex regulatory roles of physiological status and drug interactions in VCZ metabolism. Hypoalbuminemia is associated with elevated VCZ concentrations, indicating reduced clearance of the drug in patients with low protein levels, which is consistent with findings reported in the literature (Li et al., 2024; Dote et al., 2016). Lower weight and reduced platelet counts were correlated with higher VCZ exposure, indicating the overall impact of blood volume, tissue distribution, and metabolic capacity on pharmacokinetics (Huang et al., 2022; Wang et al., 2014). These parameters may serve as practical clinical tools for assessing VCZ concentration trends. Regarding drug interactions, pantoprazole, as a weak inhibitor of CYP2C19, might have its effects masked by other clinical factors. Conversely, dexamethasone, an inducer of CYP3A4, may enhance VCZ metabolism. This highlights the need for clinicians to comprehensively evaluate interactions involving multiple enzyme systems and consider patients’ metabolic phenotypes during combination therapy to mitigate potential drug interaction risks (Hirai et al., 2024; Qi et al., 2017).

As a dual-center retrospective analysis, the primary strength of this study lies in the integration of patient data from distinct medical centers, thereby enhancing sample diversity and the preliminary generalizability of the findings. However, the specific predictive performance of the identified factors (e.g., CRP, loading dose) still requires further validation and calibration in prospective, multi-center cohorts or independent datasets utilizing internal validation techniques such as cross-validation or bootstrap resampling. This constitutes a necessary next step for translating the study’s conclusions into routine clinical application.

This study has several limitations. First, due to the lack of CYP2C19 genotype data, we were unable to assess the potential interaction between genetic polymorphisms and inflammation. As a retrospective analysis, genotyping could not be performed on the enrolled patients; therefore, the observed association between C-reactive protein and voriconazole concentrations may be confounded by metabolizer phenotype. Future prospective studies should integrate genetic information with dynamic inflammatory markers to advance the development of individualized predictive models. Nevertheless, the high predictive value demonstrated by C-reactive protein still provides a simple and practical risk stratification tool for clinical settings where timely genetic testing is unavailable. Second, subsequent studies should incorporate cytokines such as interleukin-6 to more directly assess the inhibitory effect of inflammation on CYP enzyme activity. Additionally, the sample sizes for certain concomitant medications (e.g., rifampin and isoniazid) were small, which may limit statistical power and warrant cautious interpretation of the relevant results. Furthermore, the proportion of elderly patients in this study was relatively high; thus, the generalizability of the findings to younger populations needs further validation in cohorts including more young subjects. Finally, the patient cohort in this study encompassed a wide spectrum of underlying diseases, resulting in heterogeneous sources of inflammation. Although preliminary analyses indicated that C-reactive protein exhibited favorable predictive trends across different etiological subgroups, larger prospective studies with disease-specific stratification are still needed to confirm its robustness as a universal biomarker reflecting inflammatory exposure.

## 5 Conclusion

This study confirms that inflammatory status is a critical determinant of voriconazole pharmacokinetics. CRP not only serves as an independent positive predictor of trough concentrations but also demonstrates good predictive performance for supratherapeutic levels (>5 μg/mL) (AUC = 0.886). A CRP level exceeding 89.1 mg/L indicates a significantly increased risk of supratherapeutic concentrations, warranting enhanced monitoring. However, given its relatively low sensitivity, CRP should be utilized as one component of risk stratification, integrated with other high-risk factors such as low body weight and hepatic insufficiency. Furthermore, this study elucidates the “double-edged sword” effect of loading doses—while facilitating rapid attainment of therapeutic levels, they also significantly increase the risk of supratherapeutic concentrations (OR = 0.430). Clinical decision-making should be guided by risk stratification; heightened vigilance and earlier implementation of TDM are recommended when administering loading doses to patients with high inflammatory status, low body weight, or impaired liver function. It is recommended to incorporate CRP into initial dose prediction models and consider dose adjustment with early monitoring for patients presenting with high inflammatory status. By integrating inflammatory biomarkers with multidimensional clinical factors, this study provides a novel strategy for optimizing the precision of voriconazole dosing and risk management.

## Data Availability

The original contributions presented in the study are included in the article/Supplementary Material, further inquiries can be directed to the corresponding authors.
