# Supplementary material for: Factors affecting trough concentrations of voriconazole: a dual-center retrospective analysis focusing on loading dose and inflammatory state effects
Source: Front Pharmacol. 2026 Apr 23;17:1745737. doi: 10.3389/fphar.2026.1745737 (PMC13149103; doi:10.3389/fphar.2026.1745737)
Supplement: Supplementary file 1 [file Supplementaryfile1.docx]

**Supporting information**

**List of Contents**

| No | Contents | Page |
| --- | --- | --- |
| 1 | Figure S1. HPLC chromatogram of voriconazole and internal standard | S1 |
| 2 | Figure S2. Voriconazole standard curve | S2 |
| 3 | Table S1 Test results of voriconazole assay methodology | S2 |
| 4 | Table S2 Test results of voriconazole stability test | S3 |
| 5 | Table S3 Test results of voriconazole Precision and recovery test | S3 |
| 6 | Figure S3 Z-score quality control chart of voriconazole | S4 |

**Supporting information 1**

**
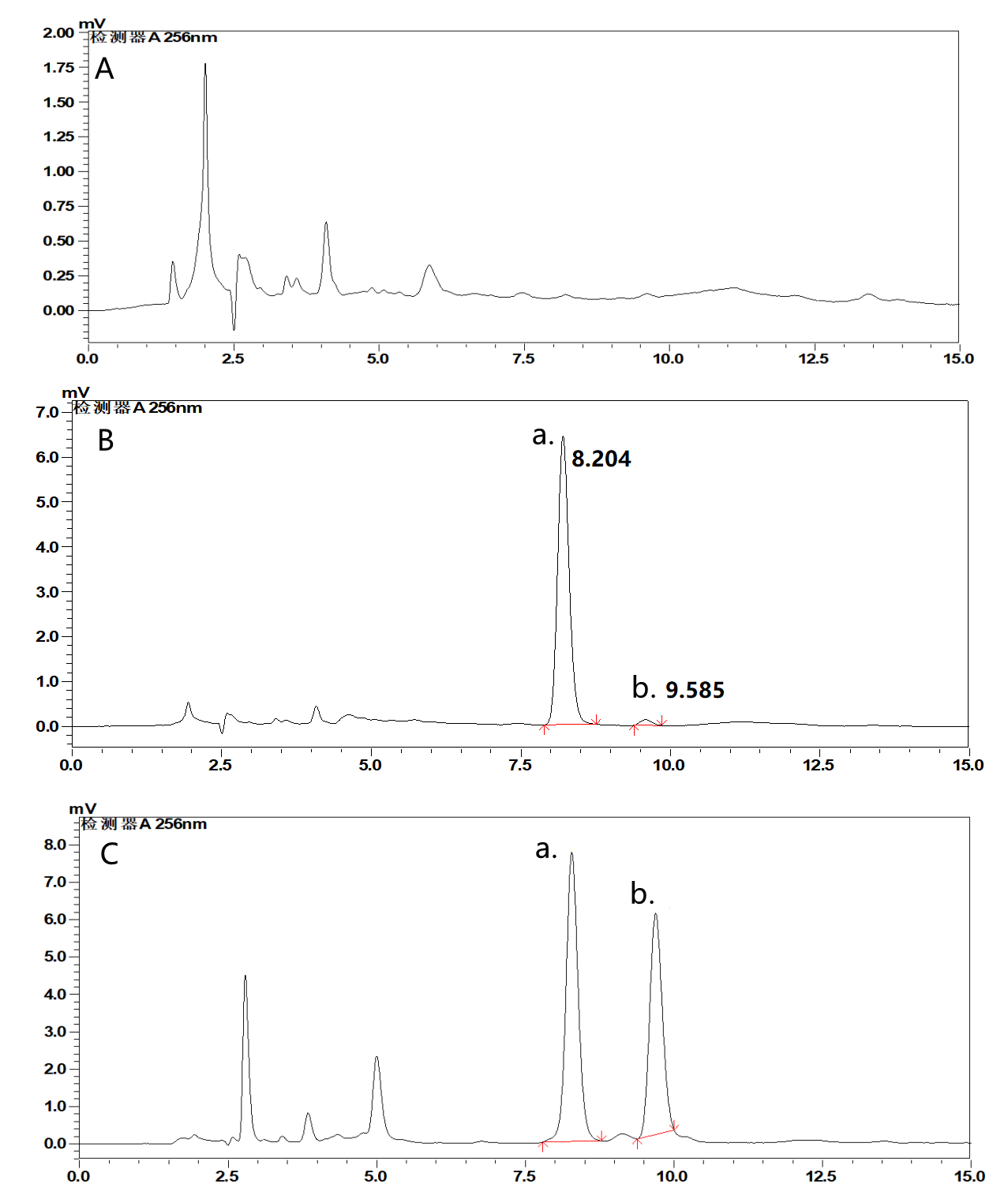
**

**Figure S1. HPLC chromatogram of voriconazole and internal standard**

Note: A blank plasma; B control; C patient plasma sample. A Internal standard peak (estazolam); B target peak (voriconazole)

**Support information 2**

**Figure S2 Voriconazole standard curve**

Note: Y = aX + b (a = 1.29795; b = 0.0031), R^2 = 0.9997481, R = 0.9998741

**Support information 3**

**Table S1 Test results of voriconazole assay methodology（n=6）**

| **Analytes** | **Concentration (μg/mL)** | | | **Precision (RSD%)** | | **Recovery** | **Matrix effect** |
| --- | --- | --- | --- | --- | --- | --- | --- |
|  | Added | Measured | Accuracy（%） | Intra-day RSD(%) | Inter-day  RSD(%) | (%, mean±SD) | (%, mean±SD) |
| VCZ | 0.3125 | 0.31±0.02 | 99.34±5.24 | 4.97 | 6.58 | 115.47±6.56 | 113.27±25.82 |
|  | 1.250 | 1.33±0.08 | 106.12±6.38 | 5.94 | 9.68 | 121.28±7.02 | 105.64±2.21 |
|  | 5.000 | 5.41±0.23 | 108.18±4.61 | 4.23 | 4.57 | 120.86±8.49 | 104.02±4.26 |

**Support information 4**

**Table S2 Test results of voriconazole stability test（n=6）**

| **Analytes** | **Stability** | | | |
| --- | --- | --- | --- | --- |
|  | Concentration  (μg/mL) | Three freeze–thaw  (%RE) | Short-term  (24 h at 25 ℃)  (%RE) | Long-term  (1 w at -20 ℃)  (%RE) |
| VCZ | 0.3125 | 0.83 | 10.25 | 6.38 |
|  | 1.250 | 7.74 | 6.55 | 1.15 |
|  | 5.000 | 5.24 | 3.40 | 0.44 |

**Support information 5**

**Table S3 Test results of voriconazole Precision and recovery test（n=6）**

| **Concentration(μg/ml)** | **Intraday precision** | | | **Daytime precision** | | |
| --- | --- | --- | --- | --- | --- | --- |
|  | **Measurement(μg/ml)** | **RSD (％)** | **recovery ratio**  **(％)** | **Measurement(μg/ml)** | **RSD (％)** | **recovery ratio**  **(％)** |
| 0.31 | 0.32±0.57 | 2.12 | 87.44 | 0.32±5.35 | 4.32 | 82.35 |
| 1.25 | 1.29±1.09 | 3.53 | 90.56 | 1.31±3.32 | 3.98 | 87.28 |
| 5.00 | 5.26±0.22 | 2.60 | 96.52 | 5.12±3.09 | 5.21 | 94.59 |

**Support information 6**

**Application of Westgard multi-rule quality control method to Indoor quality control**

According to Westgard multi-rule quality control method, the indoor quality control is to set the target value and control limit to determine the low, medium, high concentrate (1.19,3.57,5.94/μg/ml) quality control samples of voriconazole. According to the measured results, the mean value of all quality control results in one month is set as the target value. The multiple of standard deviation (SD) which include ±SD, ±2SD, ±3SD is the control limit. Because of the applicant of different concentrations with low, medium and concentration of quality control products, it is necessary to convert the measured quality control values of different concentration levels into Z fraction form and make them on a single quality control chart to compare Z fraction = (Ximat-Xmat) / Smat. Ximat is the measured value of the quality control product, Xmat is the average value of the quality control product at the same level, and Smat is the standard deviation of the quality control product.

The test results show that the errors of low, medium and high quality control samples are all in the allowable error range. Referring to the six quality control rules of Westgard multi-rule quality control method, the six quality control rules of quality control method by improving 12s/13s/22s/R4s/10x rules .The quality control rules formulated in this project are as follows: 12S shows that the measured value of a quality control product exceeds the ±2SD control limit, 13S runaway shows that the measured value of one quality control product exceeds the ±3SD control limit; 22S runaway shows that the 2 continuous control values of the same level of quality control products exceeds the +2SD or-2SD limit value in the same direction; 41S runaway shows that control values of the same level exceeds the +1SD or -1SD limit value for 4 times in a row; 7X runaway shows there are seven consecutive control values on the side of the average. Draw the Z fraction quality control chart, as shown in Figure S3. The indoor quality control evaluation system was used to evaluate the detection data to ensure the accuracy and reliability of the monitoring data of therapeutic drugs in the process of daily TDM.


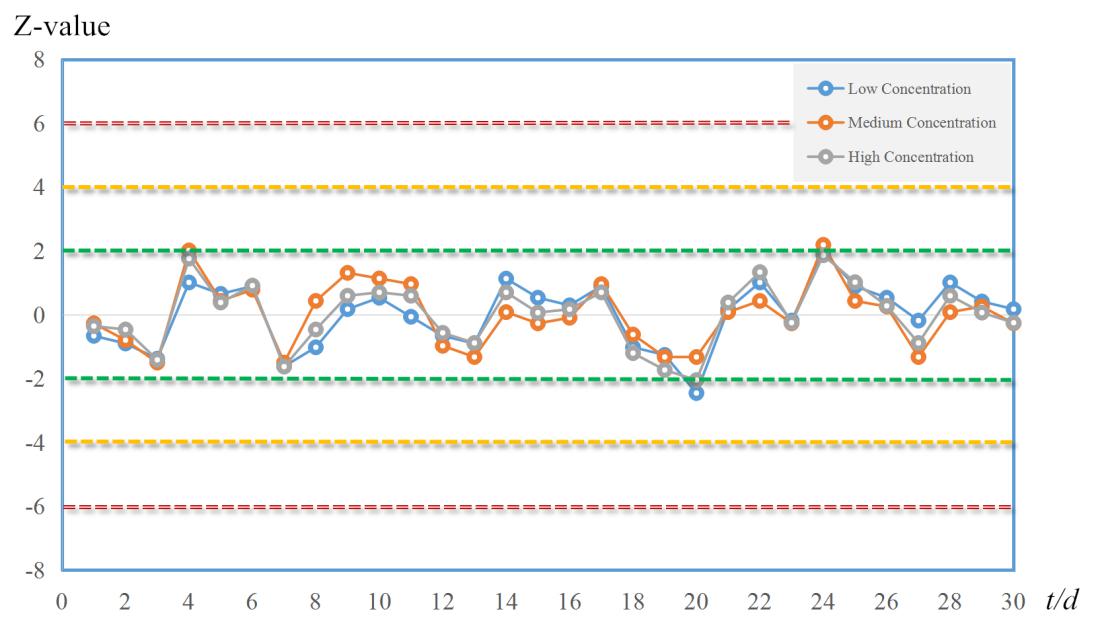


**Figure S3. Z-score quality control chart of voriconazole**
